# Real-time cardiovascular magnetic resonance at high temporal resolution: radial FLASH with nonlinear inverse reconstruction

**DOI:** 10.1186/1532-429X-12-39

**Published:** 2010-07-08

**Authors:** Shuo Zhang, Martin Uecker, Dirk Voit, Klaus-Dietmar Merboldt, Jens Frahm

**Affiliations:** 1 Biomedizinische NMR Forschungs GmbH am Max-Planck-Institut für biophysikalische Chemie, 37070 Göttingen, Germany

## Abstract

**Background:**

Functional assessments of the heart by dynamic cardiovascular magnetic resonance (CMR) commonly rely on (i) electrocardiographic (ECG) gating yielding pseudo real-time cine representations, (ii) balanced gradient-echo sequences referred to as steady-state free precession (SSFP), and (iii) breath holding or respiratory gating. Problems may therefore be due to the need for a robust ECG signal, the occurrence of arrhythmia and beat to beat variations, technical instabilities (e.g., SSFP "banding" artefacts), and limited patient compliance and comfort. Here we describe a new approach providing true real-time CMR with image acquisition times as short as 20 to 30 ms or rates of 30 to 50 frames per second.

**Methods:**

The approach relies on a previously developed real-time MR method, which combines a strongly undersampled radial FLASH CMR sequence with image reconstruction by regularized nonlinear inversion. While iterative reconstructions are currently performed offline due to limited computer speed, online monitoring during scanning is accomplished using gridding reconstructions with a sliding window at the same frame rate but with lower image quality.

**Results:**

Scans of healthy young subjects were performed at 3 T without ECG gating and during free breathing. The resulting images yield T1 contrast (depending on flip angle) with an opposed-phase or in-phase condition for water and fat signals (depending on echo time). They completely avoid (i) susceptibility-induced artefacts due to the very short echo times, (ii) radiofrequency power limitations due to excitations with flip angles of 10° or less, and (iii) the risk of peripheral nerve stimulation due to the use of normal gradient switching modes. For a section thickness of 8 mm, real-time images offer a spatial resolution and total acquisition time of 1.5 mm at 30 ms and 2.0 mm at 22 ms, respectively.

**Conclusions:**

Though awaiting thorough clinical evaluation, this work describes a robust and flexible acquisition and reconstruction technique for real-time CMR at the ultimate limit of this technology.

## Background

Cardiovascular magnetic resonance (CMR) is one of the most important, fascinating and challenging fields of clinical MR, for a recent introduction and overview see [1]. In contrast to scans of most other organs, it essentially deals with function as defined by the rapid (and not completely periodic) movements of the myocardial walls and valves, the presence of very fast (and mostly turbulent) blood flow, the status of the coronary arteries, and the perfusion of the tissue. In a technical sense, CMR acquisitions not only need to overcome motion-induced artefacts and compromised image quality in order to facilitate morphological studies, but to offensively tackle the task of a truly dynamic assessment of the functioning organ. As clearly analyzed by many authors and well known for several years, for example see [2], a solution to this problem might be offered by real-time CMR. However, despite the fact that literature searches provide numerous articles with respective keywords, so far no generally accepted approach of sufficient quality has evolved.

The purpose of this contribution is to present a novel method for real-time CMR that pushes the technology to its limits. The approach combines a radially encoded fast low-angle shot (FLASH) gradient-echo MRI technique [3] with an iterative image reconstruction by regularized nonlinear inversion. The latter algorithm was originally developed for Cartesian parallel imaging [4] and recently modified to cope with arbitrary, and in particular, radial trajectories [5]. The further extension to temporal regularization and filtering [6] resulted in an unexpected degree of data undersampling that corresponds to the acquisition of serial images with a spatial resolution of 1.5 to 2.0 mm at acquisition times of only 20 to 30 ms. This note presents the first systematic application of the new method to the heart in order to investigate the potential for an adequate visualization of cardiac movements and blood flow in healthy human subjects - during free breathing and without ECG or respiratory gating. The aim of the study focuses on a preliminary evaluation of the accessible spatiotemporal resolution and contrast as a guide for future clinical trials.

## Methods

### Subjects

Human participants with no known illness were recruited among the students of the local University. For studying the basic technical properties of the proposed real-time CMR technique we used a total of 15 subjects. All subjects gave written informed consent before each CMR scan.

### Real-time CMR

All studies were conducted at 3 T using a commercially available CMR system (Tim Trio, Siemens Healthcare, Erlangen, Germany) and a body coil for radiofrequency (RF) excitation. Subjects were examined in a supine position and CMR signals were acquired with the use of a 32-channel cardiac coil comprising an anterior and posterior 16-element array.

We used a RF-spoiled radial FLASH CMR sequence for data acquisition yielding spin-density or T1 contrast depending on the repetition time TR (here 2 - 4 ms) and flip angle of the RF excitation pulse (e.g., 2 - 12°). Variations in the gradient-echo time TE may result in opposed-phase and in-phase images with respect to intravoxel water and fat contributions. Technical details are described in [3]. In this work, the sequence was further modified to offer an interleaved multi-slice acquisition scheme (with respect to TR) which allows for the simultaneous recording of movies from multiple cross-sectional planes at the expense of temporal resolution. Moreover, the method was extended to multi-echo radial FLASH where successive gradient echoes are acquired with alternate polarity. Apart from applications that may benefit from the access to enhanced T2* contrast, this study used a dual-echo radial FLASH sequence to simultaneously record real-time CMR movies of the heart under opposed-phase and in-phase conditions.

Typically, acquisitions used 1.0 to 2.0 mm in-plane resolution (8 mm section thickness) with TR values of 2.0 ms to 3.2 ms, TE values of 1.3 ms (opposed phase) to 2.5 ms (in phase), and a flip angle of 8°. The images covered a 256 × 256 mm^2 ^FOV with a base resolution of 128 to 256 data samples. Real-time movies were obtained for rates of 20 to 50 frames per second corresponding to imaging times of 50 to 20 ms. For further details see Table 1. In general, heart movies with 20 s to 60 s duration were acquired during free breathing and without ECG or respiratory gating. Sequential scans covered the entire heart in anatomically defined orientations including short-axis views, 4-chamber views, and 2-chamber views. For real-time evaluations of 30 s duration per plane, 20 cross-sectional movies may be acquired within a total measuring time of only 10 min. Of course, the optimum approach for clinical applications needs to be determined in future studies.

**Table 1:** Typical parameters for real-time radial FLASH CMR

**Resolution/mm**^**3**^	1.0 × 1.0 × 8.0	1.5 × 1.5 × 8.0	2.0 × 2.0 × 8.0
Acquired spokes	19	15	11

Data samples/spoke	256	176	128

Field-of-view/mm	256	256	256

Repetition time/ms	2.8	2.2	2.0

Echo time/ms	1.8	1.4	1.3

Frame rate/s^-1^	20	30	45

**Acquisition time**/ms	52	33	22

### Image Reconstruction

The FLASH CMR sequence employed a radial encoding scheme with an undersampling factor of up to 25, that is the ratio of the number of spokes to π/2 × data samples per spoke. The radial data lines or spokes were acquired in an interleaved multi-turn arrangement extending over 5 successive data sets. Each single turn corresponds to a full image and contains only a very small number of spokes (typically 9 to 25). Nonlinear inverse image reconstructions were obtained from each single turn. The iterative reconstruction employed a temporal regularization with respect to the previous frame. Filtering involved a nonlinear median temporal filter that specifically exploits the properties of the interleaved radial acquisition scheme as well as an adaptive edge-preserving spatial filter. Following real-time export during scanning the data were reconstructed offline on a computer equipped with 4 graphical processing units (Tesla C1060, Nvidia, California, USA). With the current implementation reconstruction times were 2.5 s per image per graphical processing unit. The resulting images were transferred back into the databank of the CMR system in DICOM format. For further details of the reconstruction algorithm see [4-6]. Online monitoring of all acquisitions, that is immediate image reconstruction and display without any noticeable delay, was accomplished by combining the real-time data from 5 consecutive turns and reconstructing correspondingly less undersampled images by gridding with sliding-window technique as described in [3].

Although our current solution to the computational demand of a nonlinear inverse reconstruction is a "by-pass" to the host computer of the CMR system, the entire process including data transfer, image reconstruction, and image re-import is fully automated. Further progress in the parallelization of the algorithm as well as in computer hardware is expected to allow for a complete integration of the reconstruction technique into a commercially available CMR system within the foreseeable future.

## Results

**Figure 1:**
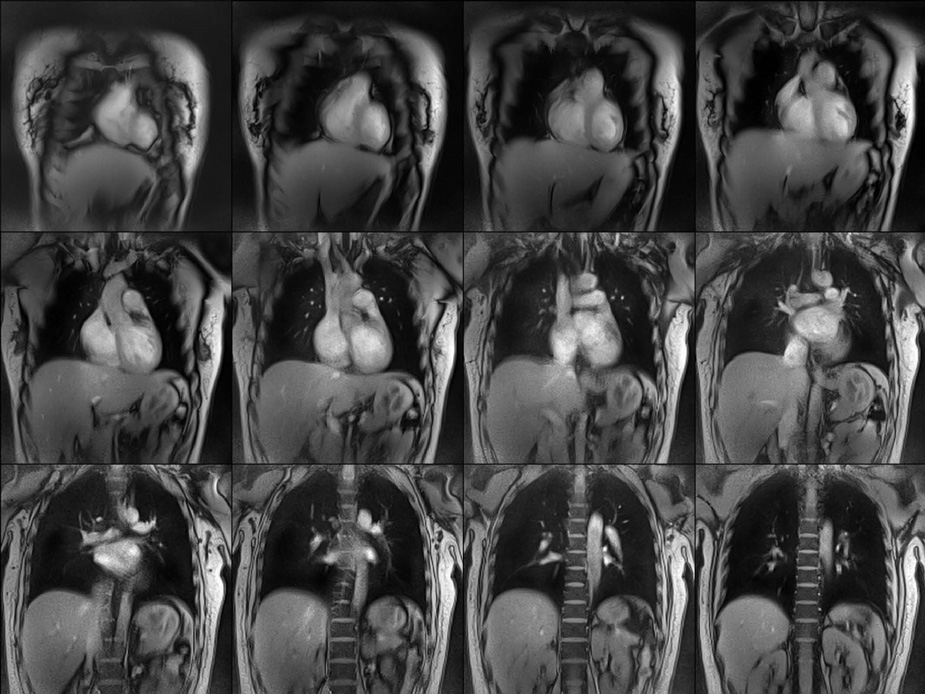
**Multislice localizer images using radial FLASH**. The serial T1-weighted images (spoiled FLASH, TR/TE = 2.2/1.4 ms, flip angle 10°) at 1.5 mm resolution, 8 mm section thickness (312 mm coronal field-of-view, free breathing, no ECG or respiratory gating), and 1000 ms acquisition time (455 spokes, 208 data samples, gridding reconstruction) demonstrate the motion robustness of radial CMR sequences and serve to plan functional assessments of the heart.

Figure 1 depicts serial T1-weighted multi-slice images of a large field-of-view that were reconstructed from fully sampled radial datasets by gridding. Despite their relatively long measuring time of 1 s, the absence of conventional (phase-encoding) artefacts confirms the well known motion robustness of radial CMR sequences. For the present study suitable sets of multi-slice images served to plan functional assessments of the heart along anatomically defined orientations without the need for an ECG signal.

**Figure 2:**
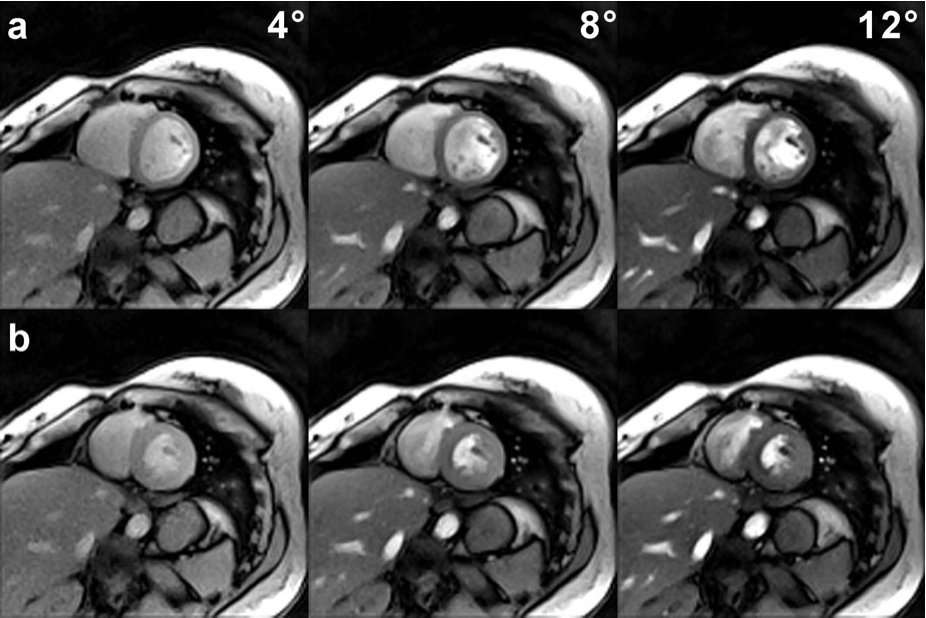
**T1 contrast in real-time radial FLASH CMR**. T1-weighted images (spoiled FLASH, TR/TE = 2.0/1.3 ms, 15 spokes, acquisition time 30 ms) with increasing T1 contrast as a function of flip angle (4°, 8°, 12°) for a short-axis view (2.0 mm resolution, 8 mm section thickness) during (a) diastole and (b) systole. The images were selected from respective real-time CMR movies reconstructed by nonlinear inversion.

The basic T1 contrast of the RF-spoiled radial FLASH images is illustrated in Figure 2 as a function of flip angle for a diastolic and systolic short-axis view. The images were taken from respective movies reconstructed by nonlinear inversion at 2.0 mm resolution and 30 ms acquisition time (TR = 2.0 ms with 15 spokes per image). They range from mild (4°) to strong (12°) T1 weighting, with 8° taken as a typical value for most subsequent investigations. Access to spin density-weighted movies may be ensured for even lower flip angles such as 2° (not shown), which lead to a vanishing contrast between blood and myocardium and also eliminate any inflow contrast.

**Figure 3:**
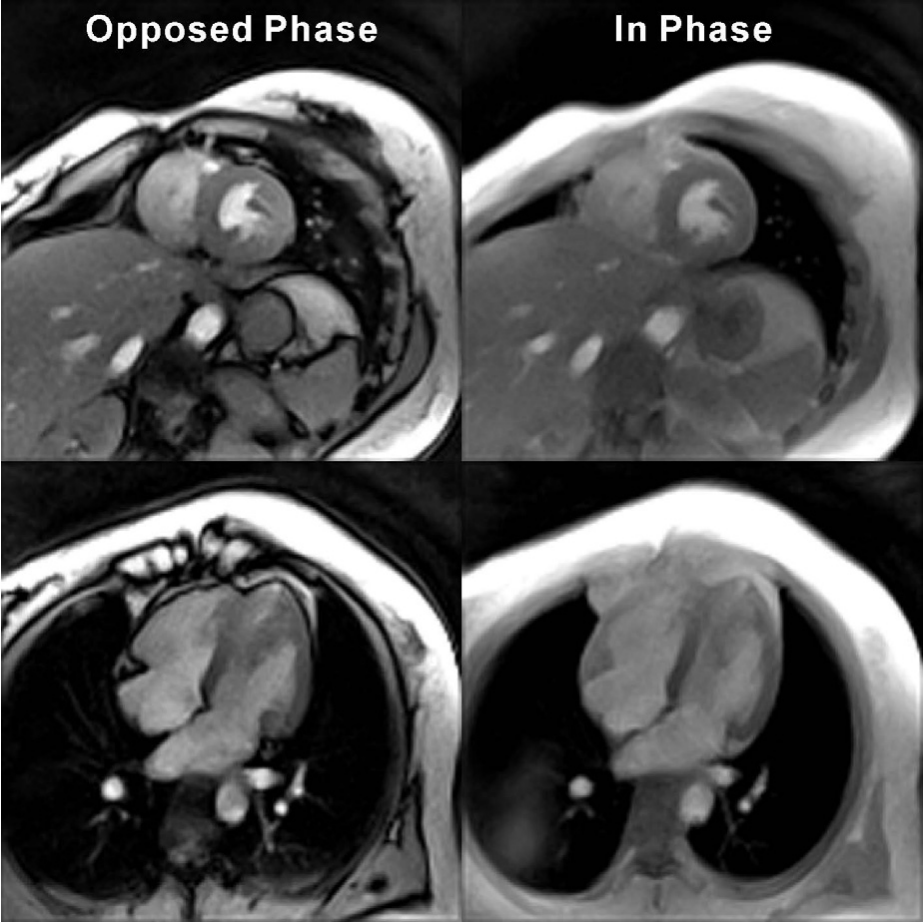
**Water and fat signals in real-time radial FLASH CMR**. Opposed-phase (spoiled FLASH, TR/TE = 2.0/1.3 ms, flip angle 8°, 15 spokes, acquisition time 30 ms) and in-phase images (TR/TE = 3.2/2.5 ms, acquisition time 48 ms) for (top) a short-axis and (bottom) a 4-chamber view (2.0 mm resolution, 8 mm section thickness). The images were selected from respective real-time CMR movies reconstructed by nonlinear inversion.

**Figure 4:**
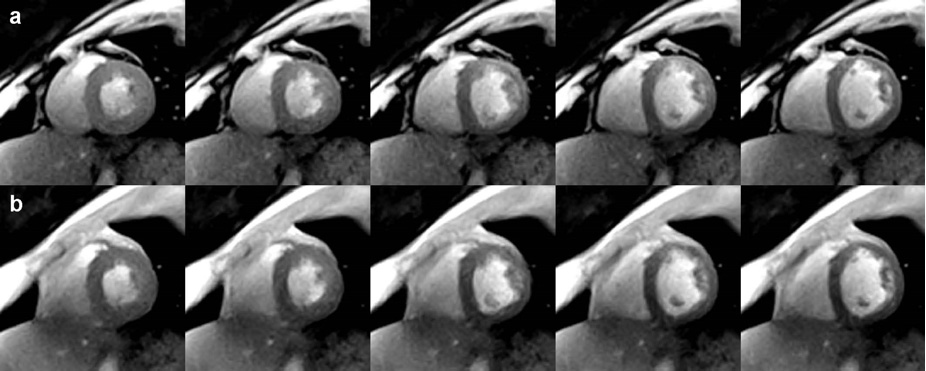
**Simultaneous opposed-phase and in-phase real-time radial FLASH CMR**. T1-weighted images (spoiled multi-echo FLASH, TR = 3.1 ms, flip angle 8°, 11 spokes) of a short-axis view with (a) an opposed-phase (TE = 1.3 ms) and (b) in-phase condition (TE = 2.4 ms) for overlapping water and fat signals (2.0 mm resolution, 8 mm section thickness, 34 ms total acquisition time). The images cover a post-systolic expansion phase by every second frame (68 ms apart). They were selected from respective real-time CMR movies reconstructed by nonlinear inversion and zoomed by a factor of 1.5.

Figure 3 depicts the difference between opposed-phase and in-phase real-time images of the heart in both a short-axis and 4-chamber view. Accordingly, water and fat signals in the same voxel either mutually cancel or constructively superimpose. In the latter case and at the expense of a slightly prolonged echo time and correspondingly longer image acquisition time, in-phase images better delineate the pericardium, which in both views becomes visible as an outer bright rim or border zone of the darker left-ventricular myocardial wall. Because of the need for a longer TR, in-phase movies may simultaneously be obtained with opposed-phase movies when using a dual-echo CMR sequence. Figure 4 presents sequential pairs of images with opposed-phase and in-phase contrast where each pair was acquired within an acquisition time of 34 ms (TR = 3.1 ms, 2 gradient echoes, 11 spokes). The frames are taken from a respective dual-echo movie in short-axis orientation at 2.0 mm resolution and 32 frames per second.

**Figure 5:**
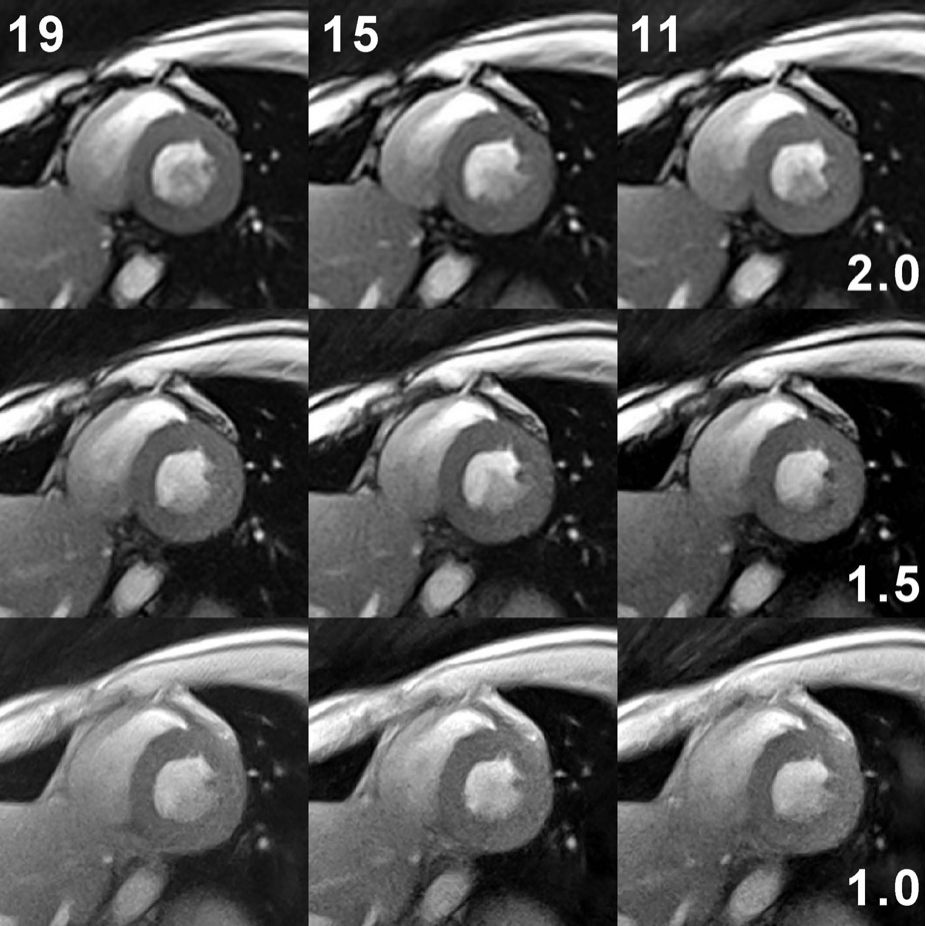
**Spatial vs temporal resolution in real-time radial FLASH CMR**. T1-weighted images (spoiled FLASH, flip angle 8°) of a short-axis view during systole at 2.0 mm (TR/TE = 2.0/1.3 ms), 1.5 mm (TR/TE = 2.2/1.4 ms), and 1.0 mm resolution (TR/TE = 2.8/1.8 ms) using data sets with only 19, 15, and 11 spokes, respectively. Individual acquisition times are given by the # spokes × TR and range from 22 ms (upper right: 11 spokes and TR = 2.0 ms) to 52 ms (lower left: 19 spokes and TR = 2.8 ms). The images were selected from respective real-time CMR movies reconstructed by nonlinear inversion and zoomed by a factor of 1.5.

**Figure 6:**
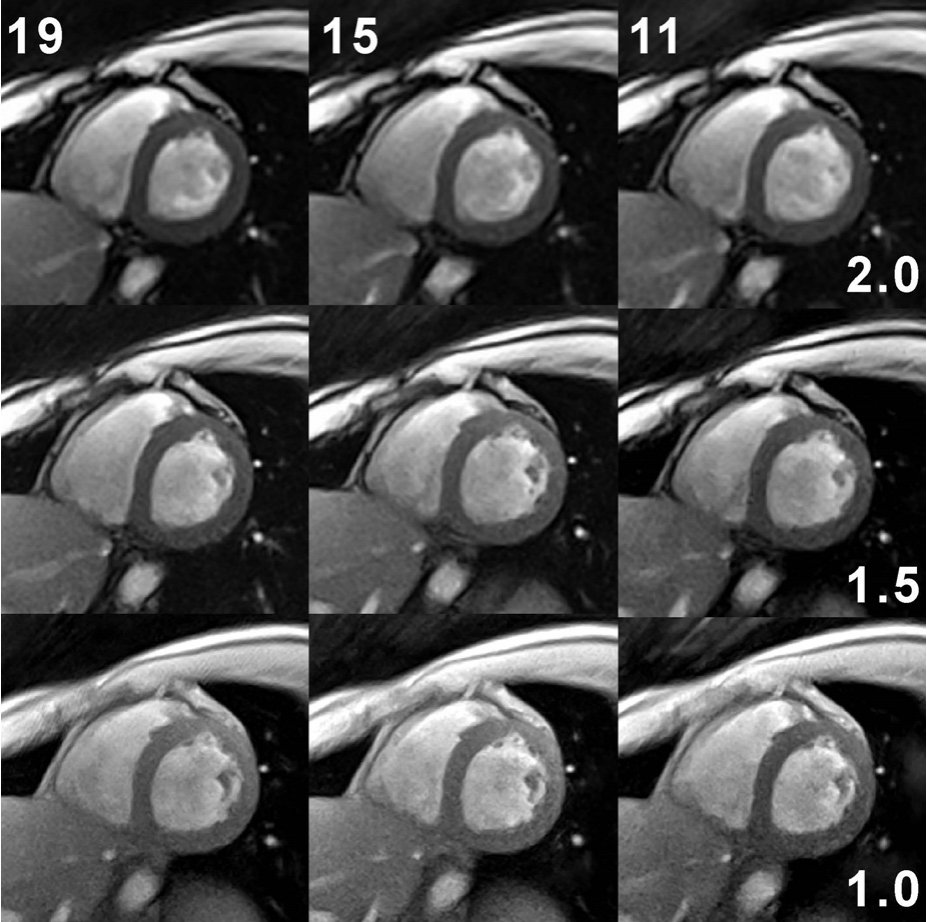
**Spatial vs temporal resolution in real-time radial FLASH CMR**. Same as Fig. 4 but for a diastolic phase.

The flexibility of the proposed real-time CMR method to trade spatial vs temporal resolution is demonstrated in Figures 5 and 6 for systolic and diastolic short-axis views, respectively. Reasonable choices with respect to overall image quality seem to be on the diagonal from the upper right to the lower left corner (see parameters summarized in Table 1). Corresponding images either emphasize speed (22 ms acquisition time for a 2.0 mm in-plane resolution) or resolution (52 ms for 1.0 mm). As a good working compromise for the experimental conditions (field strength, cardiac coil, etc) used here we consider the central images of Figures 5 and 6 with 33 ms acquisition time (30 frames per second) and 1.5 mm resolution. The images in the upper left (38 ms, 2.0 mm) and lower right corner (31 ms, 1.0 mm) tend to be affected by spatial blurring or a limited signal-to-noise ratio (SNR), respectively. In general, however, with acquisition times of 20 ms to 50 ms all images present with a high degree of temporal fidelity and do not suffer from any visible sign of temporal blurring.

**Figure 7:**
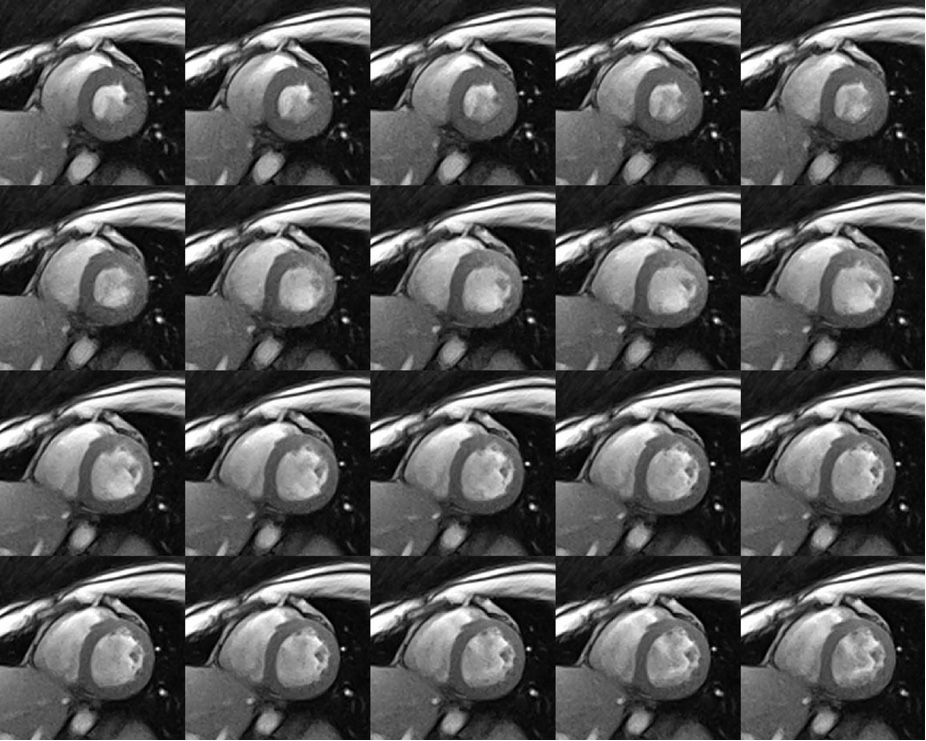
**Real-time radial FLASH CMR at 1.5 mm spatial resolution and 33 ms temporal resolution**. Consecutive T1-weighted images (spoiled FLASH, TR/TE = 2.2/1.4 ms, flip angle 8°, 15 spokes) of a short-axis view at 1.5 mm resolution, 8 mm section thickness, and 33 ms acquisition time. The 20 images cover a 660 ms period of a single cardiac cycle from systole (upper left) to diastole (lower right). They were selected from a respective real-time CMR movie reconstructed by nonlinear inversion (see Additional File 1) and zoomed by a factor of 1.5.

**Figure 8:**
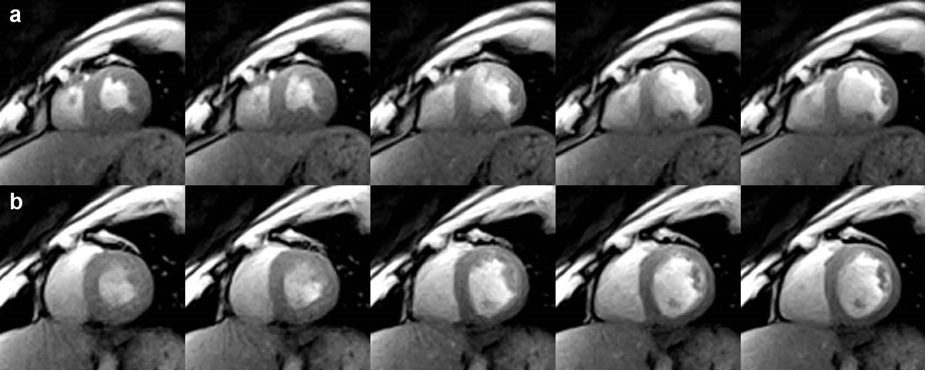
**Interleaved dual-slice real-time radial FLASH CMR**. T1-weighted images (spoiled multi-slice FLASH, TR = 4.0 ms for two sections, TE = 2.0 ms, flip angle 10°, 11 spokes, 44 ms total acquisition time) of two simultaneously acquired short-axis views (a) in the apical segment and (b) 20 mm above in the mid-cavity segment (2.0 mm resolution, 8 mm section thickness). The images cover a post-systolic expansion phase by every second frame (88 ms apart). They were selected from respective real-time CMR movies reconstructed by nonlinear inversion and zoomed by a factor of 1.5.

The sharpness of the images and the potential to characterize myocardial movements with excellent accuracy are confirmed by the 20 consecutive real-time images in Figure 7 which were taken from a single cardiac cycle of a longer movie sequence (see Additional File 1). At 1.5 mm resolution and 33 ms acquisition time these images refer to a 660 ms period from end systole as defined by maximum contraction and wall thickening to end diastole. On the other hand, the available speed of the proposed method may be exploited for the simultaneous recording of multiple cross-sectional movies. Figure 8 shows a representative example from an interleaved dual-slice acquisition at 2.0 mm resolution and 23 frames per second. Each of the selected pairs of frames was acquired within 44 ms (TR = 4.0 ms, 2 sections, 11 spokes). The series covers the post-systolic expansion of the heart in two short-axis views 20 mm apart.

**Figure 9:**
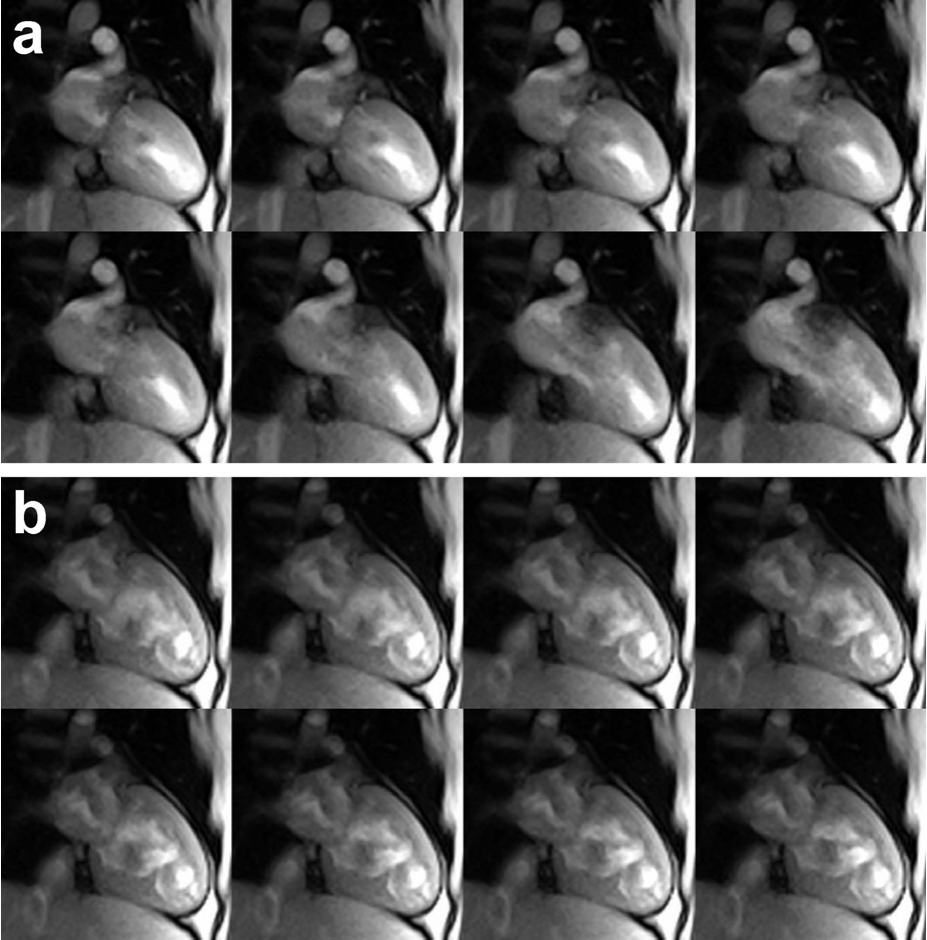
**Real-time radial FLASH CMR at 2.0 mm spatial resolution and 22 ms temporal resolution**. Consecutive T1-weighted images (spoiled FLASH, TR/TE = 2.0/1.3 ms, flip angle 8°, 11 spokes) of a 2-chamber view at 2.0 mm resolution, 8 mm section thickness, and 22 ms acquisition time. The images depict two 176 ms periods (8 images) of a single cardiac cycle that are 682 ms (31 images) apart: (a) opening of the mitral valve and resulting blood stream into the left ventricle, (b) turbulent flow leading to a vortex at the apex of the left ventricle. They were selected from a respective real-time CMR movie reconstructed by nonlinear inversion (see Additional File 2) and zoomed by a factor of 1.5.

Finally, Figure 9 demonstrates cardiovascular blood flow in the left ventricle in two different phases of the cardiac cycle (see Additional File 2). The images refer to consecutive 2-chamber views taken from a single heart beat at 22 ms temporal resolution. They depict two 176 ms periods (8 images each) which are 682 ms (31 images) apart. The first part coincides with the transient phase from end systole to diastole. It illustrates the opening of the mitral valve and the resulting blood stream from the left atrium into the left ventricle. The second part represents a period during late diastole and demonstrates the development of turbulent flow which leads to a vortex at the apex of the heart.

## Discussion

Almost 25 years ago the advent of rapid gradient-echo MRI was followed by the introduction of ECG-gated gradient-echo sequences that revolutionized CMR studies by initiating and stimulating a large range of new techniques for functional assessments. In more recent years, most dynamic evaluations seem to favour balanced steady-state free precession (SSFP) gradient-echo CMR sequences that deliberately strengthen (by high flip angles) and subsequently refocus transverse coherences (by fully symmetric gradient waveforms that zero the net phase per TR). While resulting in T1/T2-weighted CMR signal intensities, the clinical purpose is the emphasis of bright-blood contrast to facilitate the segmentation of the darkened myocardium. However, at higher magnetic field strengths such as 3 T the off-resonance sensitivity of balanced SSFP sequences usually leads to dark "banding" artefacts, which in the context of real-time CMR, that is for moving objects, vary with time and therefore can almost never be accounted for by a static correction. Other potential problems may arise from the use of very high flip angles that exceed the limits for the specific absorption rate and degrade the slice profile. Although our implementation of the radial FLASH CMR technique also allows for the use of refocusing or even fully balanced gradients [3], the much faster acquisition of T1-weighted images is expected to achieve both sufficient blood-tissue contrast and clearly visible myocardial walls. While the present observations support this notion, it certainly has to be further evaluated in true clinical settings.

For the spoiled T1-weighted version, the proposed real-time CMR method emerges as a robust and flexible approach that results in high image quality and promises very few if any failed cardiac scans. The images do not suffer from susceptibility, off-resonance, or motion artefacts, and applications are not restricted by reaching RF power deposition limits. As a consequence, the method may fully exploit the advantages of higher magnetic fields such as 3 T for CMR. As far as contrast is concerned, the sequence provides access to spin density and T1 contrast as well as in-phase and opposed-phase conditions for overlapping water and fat signal contributions. Most importantly, the presented real-time cardiac images offer high spatial resolution at so far unsurpassed temporal resolution.

Of course, this note only discusses the basic technical properties of real-time heart images in terms of spatiotemporal resolution and contrast. Therefore, the next obvious step must be the development (or adjustment) of suitable clinical protocols for the evaluation of different heart diseases. In this respect, it is worth mentioning that the proposed method may be extended in a number of ways. Apart from further improvements of the reconstruction algorithm, for example with respect to regularization and filtering, actual developments include algorithms that achieve a true water/fat image separation from multi-echo acquisitions at arbitrary echo times. Other promising variants refer to the incorporation of flow-encoding magnetic field gradients for real-time phase-contrast CMR and the development of sequential multi-slice protocols complementing the interleaved version shown here. Because interleaved procedures lower the temporal resolution by a factor given by the number of desired sections, it may be advisable to perform sequential acquisitions for a larger number of sections before continuing with a serial recording. This strategy keeps the individual image acquisition time short, but introduces a temporal gap for movies of each section that again depends on the number of sections. Although posing new problems for the regularization of the nonlinear inverse problem, sequential multi-slice movies may be a solution for perfusion studies ("late gadolinium enhancement") as they continuously cover the entire heart with still adequate temporal resolution.

Finally, in order to translate real-time CMR acquisitions into clinical assessments of myocardial function, perfusion, and viability, established measuring protocols and quantitative evaluations need to be adapted to the experimental conditions of a real-time scan. An important prerequisite is the simultaneous recording of the ECG signal to assign the relative time to the R wave to all images, similar to its current use for retrospective gating. This goal was accomplished by integrating an optional ECG time stamp into the real-time radial FLASH CMR sequence, so that most analysis tools should be applicable with only minor adjustments.

## Conclusions

Though awaiting thorough clinical evaluation, this work describes a robust and flexible acquisition and reconstruction technique for real-time CMR at the ultimate limit of this technology.

## Competing interests

The authors declare that they have no competing interests.

## Authors' contributions

SZ helped to design the study and to develop the CMR acquisition technique, performed the CMR scans, and assisted in the interpretation of the results. MU developed the mathematical procedures and performed the image reconstruction and analysis. DV helped to develop the CMR acquisition technique and in the interpretation of the results. KDM helped to develop the CMR acquisition technique, participated in the CMR scans, and assisted in the interpretation of the results. JF conceived the study and participated in its design, supervised the data acquisition, interpreted results and drafted the manuscript. All authors read and approved the final manuscript.

## Supplementary Material

Additional file 1 **Real-time CMR (short-axis view) at 1.5 mm spatial resolution and 33 ms temporal resolution**. The T1-weighted images were acquired with an RF-spoiled radial FLASH CMR sequence (TR/TE = 2.2/1.4 ms, flip angle 8°, 15 spokes) at 1.5 mm resolution, 8 mm section thickness, and 33 ms acquisition time without ECG or respiratory gating. Image reconstruction was achieved by regularized nonlinear inversion. Click here for file

Additional file 2 **Real-time CMR (2-chamber view) at 2.0 mm spatial resolution and 22 ms temporal resolution**. The T1-weighted images were acquired with an RF-spoiled radial FLASH CMR sequence (TR/TE = 2.0/1.3 ms, flip angle 8°, 11 spokes) at 2.0 mm resolution, 8 mm section thickness, and 22 ms acquisition time without ECG or respiratory gating. Image reconstruction was achieved by regularized nonlinear inversion. Click here for file
